# Implantation of an Innovative Intracardiac Microcomputer System for Web-Based Real-Time Monitoring of Heart Failure: Usability and Patients’ Attitudes

**DOI:** 10.2196/21055

**Published:** 2021-04-21

**Authors:** Giuseppe D´Ancona, Monica Murero, Sebastian Feickert, Hilmi Kaplan, Alper Öner, Jasmin Ortak, Hueseyin Ince

**Affiliations:** 1 Department of Cardiology Vivantes Hospital Am Urban Berlin Germany; 2 Federico II University Naples Italy; 3 Department of Cardiology, Rostock University Rostock Germany

**Keywords:** heart, failure, left atrial, pressure, intracardiac, device, monitoring, implantable, wireless, transmission, web-based

## Abstract

**Background:**

Heart failure (HF) management guided by the measurement of intracardiac and pulmonary pressure values obtained through innovative permanent intracardiac microsensors has been recently proposed as a valid strategy to individualize treatment and anticipate hemodynamic destabilization. These sensors have potential to reduce patient hospitalization rates and optimize quality of life.

**Objective:**

The aim of this study was to evaluate the usability and patients’ attitudes toward a new permanent intracardiac device implanted to remotely monitor left intra-atrial pressures (V-LAP, Vectorious Medical Technologies, Tel Aviv, Israel) in patients with chronic HF.

**Methods:**

The V-LAP system is a miniaturized sensor implanted percutaneously across the interatrial septum. The system communicates wirelessly with a “companion device” (a wearable belt) that is placed on the patient’s chest at the time of acquisition/transmission of left heart pressure measurements. At first follow-up after implantation, the patients and health care providers were asked to fill out a questionnaire on the usability of the system, ease in performing the various required tasks (data acquisition and transmission), and overall satisfaction. Replies to the questions were mainly given using a 5-point Likert scale (1: very poor, 2: poor, 3: average, 4: good, 5: excellent). Further patient follow-ups were performed at 3, 6, and 12 months.

**Results:**

Use and acceptance of the first 14 patients receiving the V-LAP technology worldwide and related health care providers have been analyzed to date. No periprocedural morbidity/mortality was observed. Before discharge, a tailored educational session was performed after device implantation with the patients and their health care providers. At the first follow-up, the mean score for overall comfort in technology use was 3.7 (SD 1.2) with 93% (13/14) of patients succeeding in applying and operating the system independently. For health care providers, the mean score for overall ease and comfort in use of the technology was 4.2 (SD 0.8). No significant differences were found between the patients’ and health care providers’ replies to the questionnaires. There was a general trend for higher scores in patients’ usability reports at later follow-ups, in which the score related to overall comfort with using the technology increased from 3.0 (SD 1.4) to 4.0 (SD 0.7) (*P*=.40) and comfort with wearing and adjusting the measuring thoracic belt increased from 2.8 (SD 1.0) to 4.2 (SD 0.4) (*P*=.02).

**Conclusions:**

Despite the gravity of their HF pathology and the complexity of their comorbid profile, patients are comfortable in using the V-LAP technology and, in the majority of cases, they can correctly and consistently acquire and transmit hemodynamic data. Although the overall patient/care provider satisfaction with the V-LAP system seems to be acceptable, improvements can be achieved after ameliorating the design of the measuring tools.

**Trial Registration:**

ClincalTrials.gov NCT03775161; https://clinicaltrials.gov/ct2/show/NCT03775161

## Introduction

Heart failure (HF) is a pandemic with important public health implications [1,2]. Patient management guided by the measurement of intracardiac and pulmonary pressure values, obtained through innovative permanent intracardiac microsensors, has been recently proposed as a valid strategy to individualize and anticipate the management of patients with chronic HF, with the goal of reducing their hospitalization rate and optimizing their quality of life [3-6]. In this context, the patients’ perspective on the use and acceptance of these innovative implantable technologies has been poorly studied.

We here report our experience with implantation of a new intracardiac device designed to monitor the left intra-atrial pressure (LAP) of patients with chronic HF through an internet-based information system. The applicability and effectiveness of this technology are currently under evaluation in a multicenter prospective trial (V-LAP study). We here focus on evaluation of device usability and satisfaction as perceived by both patients and health care providers.

## Methods

### Study Design

This study was developed as part of a multicenter prospective study (ClinicalTrials.gov NCT03775161) aimed at assessing the safety, usability, and performance of an intracardiac microsensor (V-LAP) implanted in patients with chronic HF that are subject to multiple rehospitalizations for acute decompensation. The trial was reviewed and approved by the ethical and scientific committees of the participating centers. All patients recruited signed an informed consent form to the processing and use of data for research purposes.

### System

**Figure 1:**
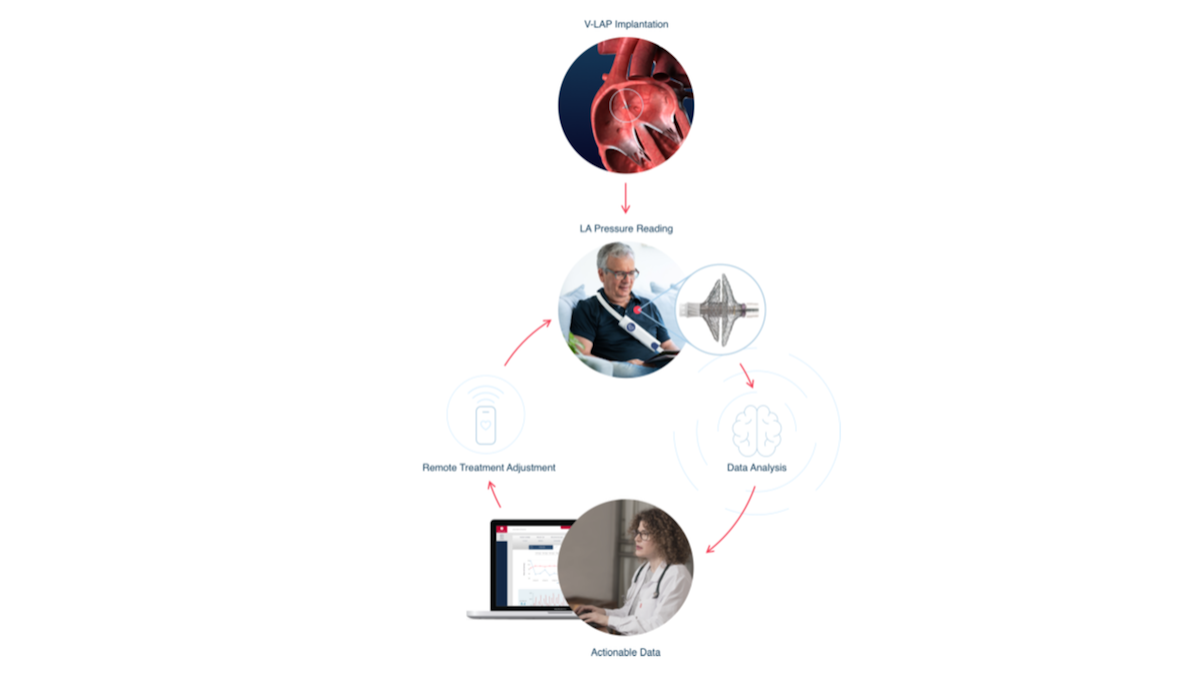
Intracardiac V-LAP Vectorious device implanted on the left side of the interatrial septum and the cycle of use.

**Figure 2:**
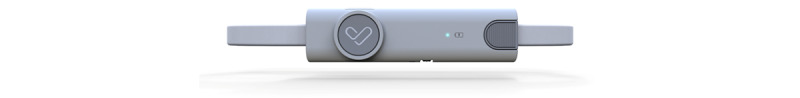
Modified low-profile thoracic belt.

**Figure 3:**
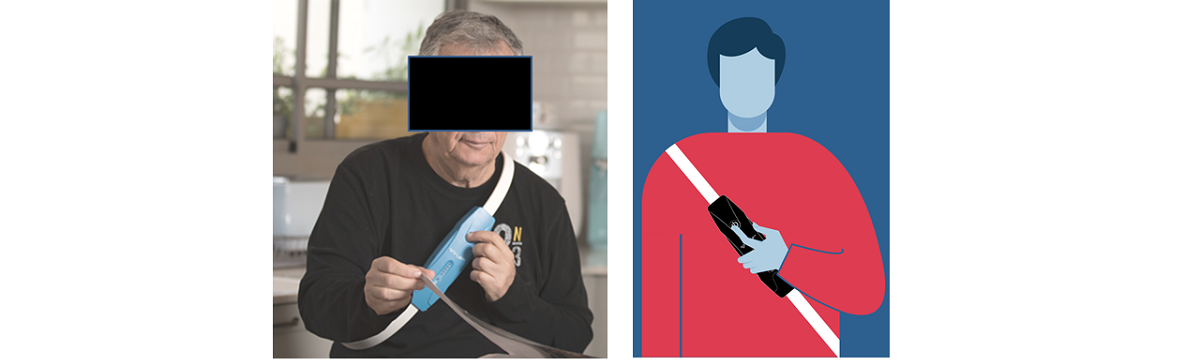
External measuring device (companion device, thoracic belt) and measurement performed after “belt” wearing.

**Figure 4:**
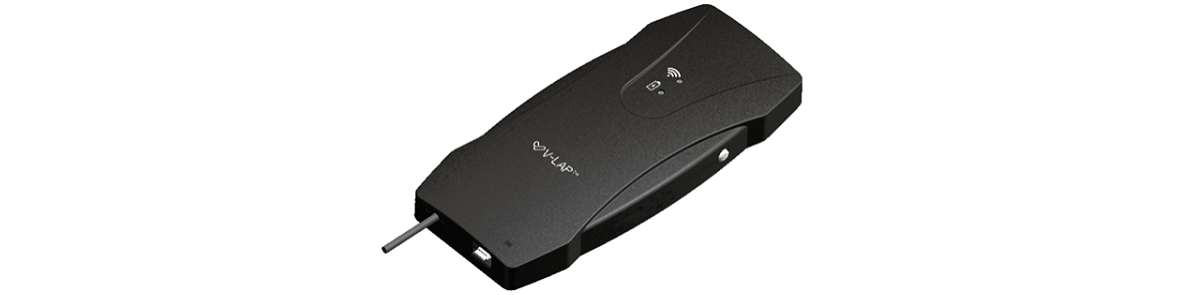
Gateway companion device.

The V-LAP system (Vectorious Medical Technologies, Tel Aviv, Israel) is the latest-generation system that enables monitoring of the patient LAP. The left atrium is the left heart chamber located directly above the left ventricle, which is the portion of the heart mainly involved in HF. The pressure inside the left atrium accurately reflects the changes in pressure within the left ventricle and can therefore be used to monitor cardiac function changes during the different phases of HF. The V-LAP system is a miniaturized sensor that is implanted completely percutaneously (ie, without incision) from the femoral vein (groin) and is anchored across the interatrial septum with the sensor portion protruding into the left atrium (Figure 1). The implant is a pressure microsensor with a low-profile design (<18 mm long and 3.9 mm in diameter) that allows for taking pressure measurements (Figure 2). The V-LAP sensory implant is fixed within the interatrial septum, usually on its thinnest area, the fossa ovalis. The implant is comprised of a hermetically sealed body that encases the sensing elements and electronics, and a nitinol braided anchor (Figure 1). The anchor has two discs, and when the implant is fully deployed, the distal and proximal discs are positioned on the left and right sides of the interatrial septum, respectively, whereas the implant body traverses the septum. The microsensor implanted inside the heart communicates wirelessly with an external system. The external system includes a lightweight, wearable, flexible sash-like loop (wearable belt companion device) that the patient can easily wear over clothing around the chest for 1-3 minutes daily (Figure 1 and Figure 3). This unit remotely powers the implant, interrogates it, and communicates LAP information to health care professionals at the HF clinic via a cellular gateway (Figure 1 and Figure 4). The external system can be used in the clinic or at any location.

After implantation, measurements are performed once or twice a day to precisely monitor the hemodynamic status of the patient.

### Predischarge Education and Usability/Satisfaction Evaluation

Before implantation of the device, the patients were informed about the technology and the implanting procedure. After implantation of the intracardiac microsensor, patients were instructed on how to independently perform daily measurements of intracardiac pressure using the external system, chest belt, and the associated gateway. Detailed information and use training were carried out on the first day after implantation. The information/educational session lasted 60 minutes and involved the patient, their health care providers (nurses and doctors specialized in the diagnosis and treatment of HF), a home care provider (whenever available), and two product technicians from the sponsor.

The session was divided into three modules: (1) familiarization with the gateway (ie, the device for power supply and data transmission to the cloud), (2) familiarization with the measuring wireless belt, and (3) appropriateness of measurement position to guarantee good communication between the internal cardiac sensor and the external measuring unit for acquisition of measurements.

An official user manual, approved by the ethics committee, was made available to both the patients and health care providers. During the first follow-up visit, 1 month after implantation, the patients and health care providers were asked to fill out a questionnaire.

The questionnaire consisted of several structured questions focusing on the usability of the system, ease in performing the various required tasks (data acquisition and transmission), and overall satisfaction. Replies to the questions were mainly given using a 5-point Likert scale (1: very poor, 2: poor, 3: average, 4: good, 5: excellent). Patient questionnaires were performed at every follow-up visit (1, 3, 6, and 12 months).

### Statistical Analysis

Data are presented as frequencies for categorical variables and as means (SD) for normally distributed continuous variables.

Scores achieved in the questionnaire responses of patients and health care providers were compared using the χ^2^ test and Student *t* test as appropriate (Likert scale results were compared considering the values as numerical) to test if the health condition and the patients’ comorbid profile impacted self-reported usability and satisfaction.

Patients’ responses at first and last follow-ups were also compared to document if usability and satisfaction improved over time. The SPSS Statistics 25 program was used for all analyses.

## Results

### Demographics and User Responses

This study focuses on the data obtained from the first 21 patients receiving the V-LAP implant worldwide (at the time of writing of this manuscript, a total of 22 patients have been treated with the implant worldwide). Table 1 shows the patient demographics and clinical data. No periprocedural complications or in-hospital mortality were observed. Usability questionnaires were completed at the time of the first follow-up visit (approximately 1 month) after discharge by the patients and their health care providers.

Table 2 shows the specific questions included in the questionnaires and the scores for each question for the 14 patients and 15 health care providers. As the study is ongoing, follow-up data of the remaining patients are still being collected.

**Table 1:** Demographic and clinical profile of the first 21 patients.

Attribute	Value
Age (years), mean (SD), range	67.0 (10.32), 49-86
Male, n (%)	17 (81)
Female, n (%)	4 (19)
Body mass index (kg/m^2^), mean (SD), range	29.44 (3.41), 24.16-36.7
CRT^a^/ICD^b^, n (%)	17 (81)
Creatinine (mg/dL), mean (SD), range (n=20)	1.55 (0.52), 0.88-2.6
eGFR^c^ (mL/min/1.73m^2^), mean (SD), range (n=19)	54.35 (20.15), 24.0-90.7
Hemoglobin (g/dL), mean (SD), range (n=20)	13.53 (1.73), 10.6-16.7
6-Minute walk (meters), mean (SD), range (n=17)	221.38 (139.15), 27.5-450.0
Saturation O_2_ (%), mean (SD), range (n=17)	96.41 (2.37), 92.0-100.0
LVEF^d^ (%), mean (SD), range (n=18)	30.78 (11.3), 15.0-55.0
Heart rate (beats/minute), mean (SD), range	72.81 (10.02), 55.0-97.0
Diastolic blood pressure (mmHg), mean (SD), range	72.1 (9.63), 55.0-92.0
Systolic blood pressure (mmHg), mean (SD), range	115.52 (14.52), 90.0-147.0
Mean RAP^e^ (mmHg), mean (SD), range (n=14)	9.29 (7.12), 1.0-22.0
PASP^f^ (mmHg), mean (SD), range (n=17)	45.0 (15.53), 6.0-68.0
Mean PCWP^g^ (mmHg), mean (SD), range (n=16)	19.38 (7.32), 8.0-37.0
LAP^h^ invasive, mean (SD), range (n=14)	18.57 (7.8), 10.0-37.0

^a^CRT: cardiac resynchronization therapy.

^b^ICD: intracardiac defibrillator.

^c^GFR: glomerular filtration rate.

^d^LVEF: left ventricular ejection fraction.

^e^RAP: right atrial pressure.

^f^PASP: pulmonary artery systolic pressure.

^g^PCWP: pulmonary capillary wedge pressure.

^h^LAP: left atrial pressure.

**Table 2:** Usability follow-up questionnaires completed by 14 patients and 15 health care providers.

Question	Patients score^a^	Health care providers score	*P* value
Success in applying and operating the system, n (%)	13 (92.9)	15 (100)	.40
Ease of wearing and fastening the belt, mean (SD)	3.7 (1.2)	4.2 (0.8)	.20
Ease of holding the belt at the appropriate measurement position, mean (SD)	3.7 (1.2)	4.2 (0.8)	.20
Ease of measurement initiation^b^, mean (SD)	4.2 (1.3)	N/A^c^	N/A
Level of comfort during measurement^b^, mean (SD)	4.0 (1.1)	N/A	N/A
Level of clarity of when the measurement is finished, mean (SD)	4.3 (1.1)	4.8 (0.4)	.10
Ease of unlocking the belt at the end of measurement^b^, mean (SD)	4.3 (1.1)	N/A	N/A
Level of clarity of when the belt needs to be charged^b^, mean (SD)	4.3 (1.2)	N/A	N/A
Ease of connecting the belt to the charger^b^, mean (SD)	4.2 (1.4)	N/A	N/A
Overall comfort and ease of use with the system, mean (SD)	3.9 (1.1)	4.2 (0.8)	.30

^a^Scores for all questions except for success in applying and operating the system were measured on a 5-point Likert scale (1: very poor, 2: poor, 3: average, 4: good, 5: excellent) 1 month after implantation.

^b^Only included in the patient questionnaire.

^c^N/A: not applicable.

### Patient Questionnaire

**Figure 5:**
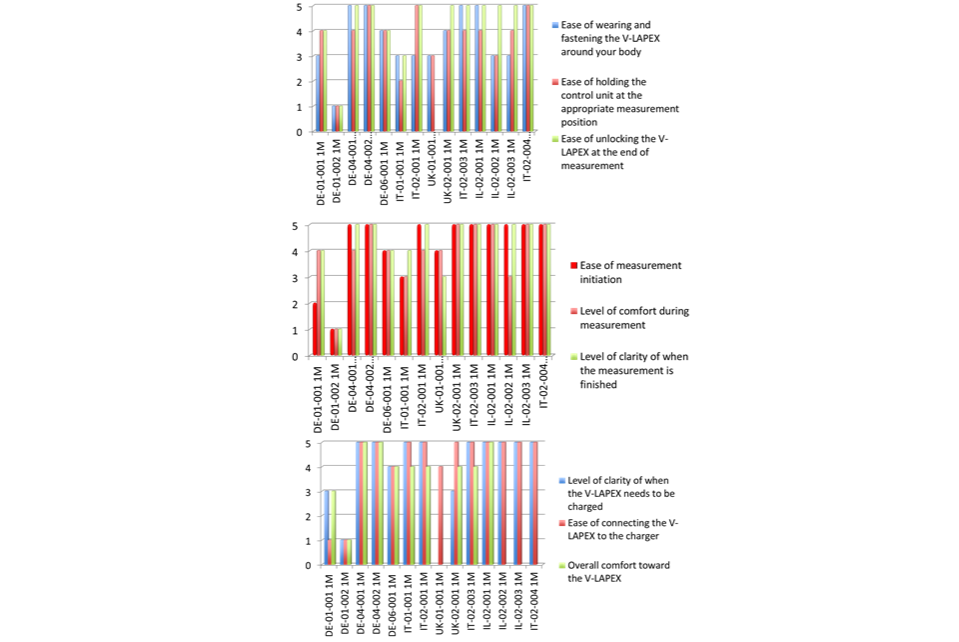
Questionnaire scores at first follow-up visit for each of the first 14 patients and each of the questions.

Figure 5 summarizes the questionnaire scores at the first follow-up visit for the first 14 patients. The overall comfort in use of the technology achieved a mean score of 3.9 at first follow-up (1 month), with 93% of the patients succeeding in applying and operating the system consistently and independently (Table 2). The lowest score was found for the ease in wearing, locking, and holding the measuring unit (belt) at the predetermined appropriate measurement position to guarantee good communication between the internal cardiac sensor and the external measuring unit (Table 2).

Patients seemed to be comfortable in starting the measurements, during the measurements, and understanding when the measurement was completed and that the belt had to be unlocked before reconnecting to the charger (Table 2 and Figure 5).

### Health Care Providers Questionnaire

All 15 health care providers included in this analysis were able to apply and operate the technology (Table 2). The mean score for overall ease and comfort in use of the technology, and for the ease in making the patient wear the thoracic belt and placing it in the appropriate measuring position reached 4.2 (Table 2). No significant differences were found between the patients’ and health care providers’ replies to the questionnaires (Table 2).

### Patient Questionnaire at First and Last Follow-Up

Table 3 summarizes the patients’ responses for each of the nine key questions at first and last follow-up. There was a general trend for higher scores of usability during follow-up, with an increase in the score for overall comfort with using the technology and specifically with wearing and adjusting the measuring thoracic belt.

**Table 3:** Patient questionnaire responses at first and last follow-up.

Question	First follow-up, mean (SD)	Last follow-up, mean (SD)	*P* value
Wearing and locking the belt	2.8 (1.0)	4.2 (0.4)	.02
Holding the belt for measurement	3.2 (1.6)	3.8 (0.8)	.50
Starting the measurement	3.0 (1.5)	4.4 (1.3)	.50
Comfort during measurements	3.2 (1.3)	3.8 (1.0)	.20
Ending measurement	3.6 (1.5)	4.2 (0.8)	.60
Unlocking the belt	3.4 (1.5)	4.2 (0.8)	.60
Charging and signal interpretation	3.2 (1.7)	3.7 (1.2)	.70
Connection to the charger	3.2 (2.0)	4.2 (1.3)	.40
Overall comfort	3.0 (1.4)	4.0 (0.8)	.40

## Discussion

### Principal Results and Comparison with Prior Work

The annual incidence of HF is increasing rapidly with an estimated worldwide prevalence of over 37.7 million people [1,2]. In its chronic phase, HF is the result of a functional cardiac insufficiency that can have multiple etiologies and manifests with numerous symptoms that compromise the patient’s quality of life. The most common symptoms of HF are shortness of breath (dyspnea), poor exercise tolerance (asthenia), and fluid retention (edema). HF is associated with significant morbidity and mortality, and confers a substantial burden on health systems in the industrialized world. Indeed, HF is the leading cause of hospitalization among adults and the elderly.

To date, the treatment of chronic HF remains predominantly reactive, focusing on drug adaptation once signs and symptoms of HF exacerbation occur. Despite continuous improvements in the long-term management of HF, patients still experience acute exacerbation of this chronic disease, resulting in recurrent hospitalization. Therefore, current HF management strategies remain inefficient in tackling hospital readmissions, and in containing morbidity and mortality. Randomized controlled trials investigating the use of external wearable technologies designed to remotely monitor patients with HF have failed to demonstrate a clear reduction in hospitalization rates [3]. The failure of these technologies may be due to the limits of the biometric parameters measured and transmitted for patient management. In fact, the telemedicine systems most widely adopted in HF management make use of sensor-based wearable devices for measuring body parameters that normally change only in a later phase of HF exacerbation. For example, changes in body temperature (as result of altered peripheral perfusion), tissue impedance (resulting from subcutaneous tissue water content), body weight (as a consequence of water retention), and urinary production (as a consequence of reduced renal perfusion) are all delayed markers of HF exacerbation.

Given the inability of noninvasively accumulated data to help in preventing hospitalizations, it has become necessary to make a paradigm shift in the use of chronic HF management strategies. As part of this shift, it is essential to integrate innovative information and communication technologies that can identify early precursors of the forthcoming exacerbation of stable HF, even if an invasive microcomputer implantation procedure may be required. Clinical evidence shows that pulmonary and intracardiac pressure increases for up to several weeks before the onset of decompensated HF symptoms. Although the CHAMPION trial showed efficacy of an implantable pulmonary artery pressure sensor to manage HF patients at risk for rehospitalizations [4], having direct measurements of left heart pressure adds sensitivity for patients affected by HF and with additional cardiac conditions [3,5,6]. In particular, the use of permanent intracardiac microsensors can detect changes in cardiac function accurately and in advance of exacerbation requiring rehospitalization. In this way, appropriate treatment can be optimized and carried out quickly, anticipating the onset of symptoms.

Correct and early acquisition of intracardiac pressure can justify prompt interventions to be undertaken at preventing hospitalizations for exacerbation of HF. Implantable intracardiac sensors allow for the remote acquisition, measurement, and analysis of patients’ meaningful data in real time. Although these technologies, as well as data derived from their use obtained to date, have generated and will continue to generate broad attention, their effectiveness and application in everyday life will depend on adequate acceptance and adoption by the treated patients. Despite substantial effort in developing and optimizing these devices, understanding the treated patients’ perspective and perception is crucial to guarantee the smooth and constant application of these costly technologies, as well as their further improvement. To the best of our knowledge, this study is the first to dedicate specific attention to patient satisfaction and ease of use after implantation of an intracardiac device for HF monitoring. For this reason, specific comparison with previous literature cannot be performed.

The main finding emerging from this study is that, despite the gravity of their HF pathology and the complexity of their comorbid profile, patients are comfortable in using the V-LAP technology and, in the majority of cases, they can correctly and consistently acquire and transmit hemodynamic data. It must be noted that before inclusion in the trial and implantation of the V-LAP technology, patients had been adequately selected, evaluating not only their clinical profile but also their psychological status, along with their attitudes toward the disease and the possible medical and behavioral measures to be undertaken to reduce hospitalization, morbidity, and mortality. In this context, our findings cannot be generalized to the plethora of patients affected by chronic HF, many of whom have difficulties in using mobile devices or performing even the simplest of daily activities. Moreover, a patient-tailored educational session was provided after device implantation with the participation of all present and future actors involved in patient management. The educational session was structured to train patients and their respective health care providers on the use of the technology and to test the correct application of the taught modules during the days of hospitalization after device implantation. Although future technological improvements will possibly lead to simplification of the patient/health care provider tasks, the continuous and direct involvement and support of the devices’ manufacturing companies should be envisaged. This can further guarantee adequate education and training of an increasing number of treated patients and of the health care providers involved in their management.

Because perceived ease of use is one of the most important factors that can increase the adoption of mobile health systems [7], a critical appraisal should be given to our findings. Despite the overall comfort in adopting the V-LAP technology, interviewed patients and respective health care providers reported the lowest scores when assessing the ease in wearing and fastening the thoracic belt and in consistently finding its appropriate position for ideal measurements. Multiple iterations are performed during hospitalization and before discharge to determine the thoracic belt’s most appropriate position allowing for optimal wireless/radiofrequency communication with the intracardiac sensor. Once the best position is identified, a picture is taken that is given to both the patient and health care provider as reference for future measurements. Frequent changes of the heart position within the chest cavity may be necessary due to physiologic and pathologic variations in cardiac hemodynamics and geometries, particularly in patients affected by HF. These variations will reflect upon the position of the intracardiac sensor, and consequently upon the wireless interaction between the external belt and intracardiac sensor, ultimately influencing the eventual sequence of signal transmission/detection.

Based on our findings, firmware version improvements have been developed and implemented by the sponsor, and a new mechanical design of the thoracic belt will be available in the very near future. The new design of the external system takes into consideration the patients’ challenges in securely fastening the belt connector to allow for continuous and uninterrupted communication with the intracardiac implant.

Moreover, and most importantly, a major improvement that is currently in development will resolve the challenges encountered in reproducing the exact positioning of the belt around the patient’s chest to guarantee adequate communication with the intracardiac implant. The new design of the companion belt has a smaller profile that will enhance placement around the patient’s chest, and allow for intuitive and precise placement in the predetermined position (Figure 3).

Adequate involvement of health care providers is crucial to guarantee the success of newly introduced and innovative technologies for monitoring HF patients. In this context, health care–related wearable and implantable technologies alone will not have the desired effect on patients’ health status improvement. In fact, data collected from these devices need to be interpreted and used within previously structured frameworks, allowing for solid and continuous interactions among patients and health care providers [3]. Interestingly, in spite of possible differences in age, health status, and digital/technology literacy between patients and health care providers, we did not find any significant difference in ease and comfort of use of the V-LAP technology. Although we are aware of the tremendous improvements already achieved with the V-LAP technology to treat HF, we do believe that a few challenges need to be overcome with the aim of further minimizing the path for data collection, transmission, analysis, and therapy adjustments. In particular, we foresee that the next generation of intracardiac monitoring systems should allow for hemodynamic and clinical data to be collected automatically without the need for actual measurements to be taken by the patient. As clinicians, we envision the possibility of an autopowered intracardiac sensor that will independently detect and transmit information about the patient’s hemodynamic status while performing their daily activities. In this light, emerging automatized systems of mobile health management based on the upcoming Internet of Things capabilities should be envisaged to increase the number of potential users accessing state-of-the-art technology. Although the average age of the currently most affected patients concerns a generation that is often technologically illiterate, it seems realistic that in only a few years, the coming generations of HF patients will find the handling of digital medical devices a matter of course and an uncomplicated task.

Finally, although beyond the scope of this study, we can confirm that V-LAP technology has immediate clinical applicability by supporting actual HF therapy changes. After observing variations in the patients’ intracardiac hemodynamic measurements taken from home, therapy adjustments were promptly updated by the remote health care providers to reflect the real-time condition, thereby avoiding hospitalization. This has been particularly useful during the ongoing COVID-19 pandemic, reducing the risk of contagion of these very fragile patients during their travel to the hospital or within the premises of outpatient clinics [8,9].

At present, patients do not receive direct feedback about the value of their own collected intracardiac data. In the near future, a companion app elaborating body-sensing data through artificial intelligence and machine learning systems may inform not only health care providers but also patients and their caregivers by offering information about the patient’s health status, proposing customized psychological comfort, and sending notifications and reminders aimed at the optimization of HF therapy. In fact, automated protocols based on artificial intelligence and machine learning are already transforming the management of other chronic diseases such as diabetes mellitus [10] and could eventually be used to maximize the potential of the V-LAP technology in the automated treatment of HF patients. In this context, it should also be emphasized that although the V-LAP system is currently mainly used to detect LAPs, correct and automated interpretation of the recorded LAP curves will facilitate the real-time monitoring of additional cardiac parameters such as the heart rhythm and mitral valve function.

### Limitations

The main limitation of this study is the small number of patients involved at present. It should be kept into consideration that the discussed technology has been only very recently introduced and is still under evaluation in a clinical trial. In fact, the patients analyzed in this study represent the majority of all patients receiving the V-LAP device worldwide.

Moreover, as emphasized above, results in terms of usability and adoption of the technology may be biased by the adequate selection of patients as part of the trial’s inclusion and exclusion protocol, which involved evaluating their psychological status, attitude toward the disease and its management, and their desire for being involved with this innovative technology. Finally, because the primary and secondary objectives of the trial were not usability and satisfaction, the sample could not be adequately sized to draw definitive conclusions on these two matters.

### Conclusions

Despite the gravity of their HF pathology and the complexity of their comorbid profile, patients are comfortable in using the V-LAP technology and, in the majority of cases, they can correctly and consistently acquire and transmit hemodynamic data. The overall patient/care provider satisfaction with the V-LAP system seems to be high. The scores of patients and respective health care providers were in the range of average to good with respect to assessing the ease in performing simple but crucial tasks such as wearing and fastening the thoracic belt, and more specifically in consistently finding its appropriate position for ideal measurements. Improvements in the external thoracic belt design have been very recently introduced and will hopefully further optimize patients’ and health care providers’ acceptance and adoption of this technology.

## Abbreviations

HF: heart failure
LAP: left intra-atrial pressure
